# Effects of 2 years of caloric restriction on oxidative status assessed by urinary F2‐isoprostanes: The CALERIE 2 randomized clinical trial

**DOI:** 10.1111/acel.12719

**Published:** 2018-02-09

**Authors:** Dora Il'yasova, Luigi Fontana, Manjushri Bhapkar, Carl F. Pieper, Ivan Spasojevic, Leanne M. Redman, Sai Krupa Das, Kim M. Huffman, William E. Kraus, the CALERIE Study Investigators

**Affiliations:** ^1^ School of Public Health Georgia State University Atlanta GA USA; ^2^ Department of Medicine Washington University School of Medicine St Louis MO USA; ^3^ Department of Clinical and Experimental Sciences Brescia University School of Medicine Brescia Italy; ^4^ CEINGE Biotecnologie Avanzate Napoli Italy; ^5^ Duke Clinical Research Institute Duke School of Medicine Duke University Durham NC USA; ^6^ Department of Biostatistics and Bioinformatics Duke University School of Medicine Durham NC USA; ^7^ Division of Medical Oncology Department of Medicine Duke Cancer Institute Duke University School of Medicine Durham NC USA; ^8^ Pennington Biomedical Research Center Baton Rouge LA USA; ^9^ Jean Mayer USDA Human Nutrition Research Center on Aging at Tufts University Boston MA USA; ^10^ Divisions of Rheumatology and Immunology and Duke Molecular Physiology Institute Duke School of Medicine Duke University Durham NC USA; ^11^ Duke Molecular Physiology Institute and Duke Clinical Research Institute Duke School of Medicine Duke University Durham NC USA

**Keywords:** caloric restriction, oxidative stress, randomized controlled trial

## Abstract

Calorie restriction (CR) without malnutrition slows aging in animal models. Oxidative stress reduction was proposed to mediate CR effects. CR effect on urinary F2‐isoprostanes, validated oxidative stress markers, was assessed in CALERIE, a two‐year randomized controlled trial. Healthy volunteers (*n *= 218) were randomized to prescribed 25% CR (*n *= 143) or ad libitum control (AL,* n *= 75) stratifying the randomization schedule by site, sex, and BMI. F2‐isoprostanes were quantified using LC‐MS/MS in morning, fasted urine specimens at baseline, at 12 and 24 months. The primary measure of oxidative status was creatinine‐adjusted 2,3‐dinor‐iPF(2α)‐III concentration, additional measured included iPF(2α)‐III, iPF2a‐VI, and 8,12‐iso‐iPF2a‐VI. Intention‐to‐treat analyses assessed change in 2,3‐dinor‐iPF(2α)‐III using mixed models assessing treatment, time, and treatment‐by‐time interaction effects, adjusted for blocking variables and baseline F2‐isoprostane value. Exploratory analyses examined changes in iPF(2α)‐III, iPF(2α)‐VI, and 8,12‐iso‐iPF(2α)‐VI. A factor analysis used aggregate information on F2‐isoprostane values. In CR group, 2,3‐dinor‐iPF(2α)‐III concentrations were reduced from baseline by 17% and 13% at 12 and 24 months, respectively; these changes were significantly different from AL group (*p *< .01). CR reduced iPF(2α)‐III concentrations by 20% and 27% at 12 and 24 months, respectively (*p *< .05). The effects were weaker on the VI‐species. CR caused statistically significant reduction in isoprostane factor at both time points, and mean (se) changes were −0.36 (0.06) and −0.31 (0.06). No significant changes in isoprostane factor were at either time point in AL group (*p *< .01 between‐group difference). We conclude that two‐year CR intervention in healthy, nonobese men and women reduced whole body oxidative stress as assessed by urinary concentrations of F2‐isoprostanes.

## 1 INTRODUCTION

Caloric restriction (CR) without malnutrition is the most powerful known intervention to slow aging and protect against cancer, heart disease, type 2 diabetes, and cognitive decline in many eukaryotic organisms, particularly rodents and monkeys (de Cabo, Carmona‐Gutierrez, Bernier, Hall & Madeo, 2014; Fontana, Partridge & Longo, 2010; Mattison et al., 2017). Multiple mechanisms have been hypothesized to mediate these beneficial effects of CR on aging, including a reduction in oxidative stress (de Cabo et al., 2014; Fontana et al., 2010). In rodents, CR decreases markers of oxidative stress and damage in multiple tissues (Sohal & Weindruch, 1996; Walsh, Shi & Van Remmen, 2014). However, there are currently no data from randomized controlled trials in humans evaluating the long‐term effects of CR on reliable markers of oxidative stress.

Oxidative stress and the accumulation of oxidative injury are deeply implicated in the pathogenesis of multiple age‐associated chronic diseases, including heart disease, type 2 diabetes, cancer, and neurodegeneration (Valko, Jomova, Rhodes, Kuca & Musilek, 2016). Urinary F2‐isoprostanes generated from polyunsaturated fatty acids constitute the validated index of oxidative damage in animal models, and the most reliable markers of nonenzymatic lipid peroxidation in vivo in humans (Basu, 2008; Il'yasova, Scarbrough & Spasojevic, 2012; Il'yasova et al., 2010; Kadiiska et al., 2005; Milne, Musiek & Morrow, 2005). High urinary F2‐isoprostane concentrations are found in patients with type 2 diabetes, hypercholesterolemia, coronary artery disease, and obesity (Basu, 2008; Valko et al., 2016). The purpose of CALERIE study—a 2‐year multicenter randomized controlled trial (RCT)—was to evaluate the effects of a prescribed 25% CR diet in healthy, nonobese young, and middle‐aged individuals on proposed longevity biomarkers and CR‐induced metabolic adaptations. One of the targeted biomarkers was urinary F2‐isoprostanes, well‐characterized and stable markers of oxidative stress (Zhang et al., 2010). In an animal study (Ward et al., 2005) and in a short‐term intervention (Buchowski et al., 2012), F2‐isoprostanes were reduced in response to CR. We measured urinary concentration of 2,3‐dinor‐iPF(2α)‐III, as the primary indicator of oxidative status, and in addition to three F2‐isoprostane isomers: iPF(2α)‐III, iPF2a‐VI, and 8,12‐iso‐iPF2a‐VI. Our primary hypothesis for this analysis—caloric restriction reduces systemic levels of oxidative damage, specifically lipid peroxidation—was based on the effects of long‐term caloric restriction in laboratory animals and/or shorter‐term human trials (Heilbronn et al., 2006; Sohal & Weindruch, 1996; Walsh et al., 2014). 2,3‐dinor‐iPF(2α)‐III was selected as the primary measure of oxidative stress, because this is a beta‐oxidation metabolite of the most frequently measured isomer iPF(2α)‐III (Basu, 2008; Milne et al., 2005). Selection of 2,3‐dinor‐iPF(2α)‐III was also based on a theoretical concern that renal tissues may contribute disproportionally to the total production of iPF(2α)‐III, whereas beta‐oxidation occurs predominantly in the extra‐renal tissues; the measurements of the beta‐oxidation metabolites may have circumvented this problem. Also, urinary concentrations of 2,3‐dinor‐iPF(2α)‐III are several fold greater as compared to its precursor iPF(2α)‐III, thus reducing measurement error. Measurements of multiple F2‐isoprostane species provided the opportunity to assess the effect of CR intervention on an aggregate F2‐isoprostane measure through factor analysis.

## 2 RESULTS

The study design has been previously reported (Rochon et al., 2011) and the main findings have been published (Ravussin et al., 2015). As previously reported, the study population was predominantly white, and approximately 70% women (Table 1). According to the enrollment criteria, mean BMI was slightly above the threshold for overweight; mean age was approximately 38 years (Table 1). At baseline, there was no difference in urinary creatinine or urinary F2‐isoprostane concentrations between the CR and AL groups.

**Table 1:** Demographic, anthropometric, and clinical characteristics at baseline for the 218 participants who started the 2‐year intervention

	Ad libitum (*n *= 75)	Caloric restriction (*n *= 143)
	N (%)	
Sex		
Female	53 (70.7)	99 (69.2)
Male	22 (29.3)	44 (30.8)
Race		
White	57 (76)	111 (77.6)
African American	11 (14.7)	15 (10.5)
Other	7 (9.3)	17 (11.9)

	Mean (*SD*)	
Age, year	37.9 (6.94)	38.0 (7.34)
BMI, kg/m^2^	25.1 (1.64)	25.2 (1.78)
Creatinine, mg/ml	0.613 (0.224)	0.720 (0.350)
Urinary F2‐isoprostanes, ng/mg creatinine		
Primary measure		
2,3‐dinor‐iPF(2α)‐III	1.90 (0.86)	1.89 (0.81)
Secondary measures		
iPF(2α)‐III	0.211 (0.138)	0. 209 (0.124)
iPF(2α)‐VI	4.05 (1.55)	4.12 (1.80)
8,12‐iso‐iPF(2α)‐VI	5.35 (2.35)	5.43 (2.41)

AL, ad libitum control group; CR, 25% caloric restriction group.

We examined crude cross‐sectional associations between urinary F2‐isoprostanes and participant characteristics known to be associated with these biomarkers in other study populations (Table 2) (Il'yasova, Wang, et al., 2012; Kanaya et al., 2011; Keaney et al., 2003). As expected, urinary F2‐isoprostane concentrations were greater in women, with the least female–male difference being in 8,12‐iso‐iPF(2α)‐VI (not statistically significant). Overall, F2‐isoprostane concentrations tended to be lower in African American participants compared to Caucasians (Table 3). There was no correlation of urinary F2‐isoprostanes with age or BMI (Table 2).

**Table 2:** Baseline Urinary F2‐isoprostane Levels and Characteristics of CALERIE participants (*n *= 218)

	2,3‐dinor‐iPF2(2α)‐III	iPF(2α)‐III	iPF(2α)‐VI	8,12‐iso‐iPF(2α)‐VI
	Mean (*SD*) *p*‐value for Wilcoxon test			
Sex				
Females	2.10 (0.86)	0.238 (0.139)	4.47 (1.84)	5.55 (2.57)
Males	1.41 (0.47)	0.145 (0.630)	3.25 (0.97)	5.06 (1.85)
	*p *< .0001	*p *< .0001	*p *< .0001	*p *= .55
Race/ethnicity				
Caucasian	1.89 (0.84)	0.215 (0.133)	4.15 (1.82)	5.58 (2.51)
African American	1.94 (0.94)	0.187 (0.132)	3.82 (1.41)	4.26 (1.29)
Other	1.86 (0.55)	0.196 (0.990)	4.04 (1.22)	5.34 (2.06)
	*p *= .86	*p *= .28	*p *= .58	*p *= .02

	Spearman correlation coefficient *p*‐value			
Age	−0.1125 *p *= .10	−0.0429 *p *= .53	−0.0360 *p *= .60	−0.0691 *p *= .31
BMI	0.0365 *p *= .59	−0.0008 *p *= .99	−0.0125 *p *= .86	0.0757 *p *= .27

**Table 3:** Adjusted Changes in Urinary F2‐isoprostane Levels^a^ at 12 and 24 Months from Baseline by Treatment Group

ng/mg creatinine	AL *N *= 75	CR *N *= 143	*p* value AL vs. CR	P (time × treatment)
Primary analysis				
2,3‐dinor‐iPF(2α)‐III				
Change at 12 months	−0.041 ± 0.059	−0.313 ± 0.045	.0001	0.833
Change at 24 months	0.018 ± 0.075	−0.237 ± 0.058	.006	
Exploratory analyses				
iPF(2α)‐III				
Change at 12 months	−0.012 ± 0.012	−0.042 ± 0.009	.04	0.486
Change at 24 months	−0.016 ± 0.011	−0.057 ± 0.009	.003	
iPF(2α)‐VI				
Change at 12 months	−0.171 ± 0.140	−0.461 ± 0.105	.08	0.846
Change at 24 months	0.115 ± 0.157	−0.213 ± 0.121	.09	
8,12‐iso‐iPF(2α)‐VI				
Change at 12 months	−0.303 ± 0.170	−0.751 ± 0.128	.058	0.428
Change at 24 months	−0.381 ± 0.184	−0.663 ± 0.141	.42	
Isoprostane Factor				
Change at 12 months	−0.098 (0.075)	−0.358 (0.057)	.004	0.974
Change at 24 months	−0.048 (0.078)	−0.311 (0.060)	.006	

a Adjusted for site, sex, BMI, and baseline values of F2‐isoprostanes

The ITT analysis of the intervention effect showed that the reduction in 2,3‐dinor‐iPF(2α)‐III concentrations was significantly greater in the CR as compared to the AL group at both time points, 12 and 24 months (Table 3, Figure 2). Specifically, 2,3‐dinor‐iPF(2α)‐III levels decreased at 12 months with eightfold greater change in the CR group (*p *= .0001). At 24 months, 2,3‐dinor‐iPF(2α)‐III concentrations reverted to baseline in both groups: In the AL group, the concentrations were not different from baseline; in contrast, 2,3‐dinor‐iPF(2α)‐III concentrations in the CR group remained significantly reduced from baseline (*p *= .006). CR also significantly reduced urinary concentrations of iPF(2α)‐III at 12 and 24 months (*p*‐values .04 and .003, respectively); because this is the most frequently studied F2‐isoprostane species, the observed effect on iPF(2α)‐III concentrations provides a reference to previously published F2‐isoprostane studies.

**Figure 1:**
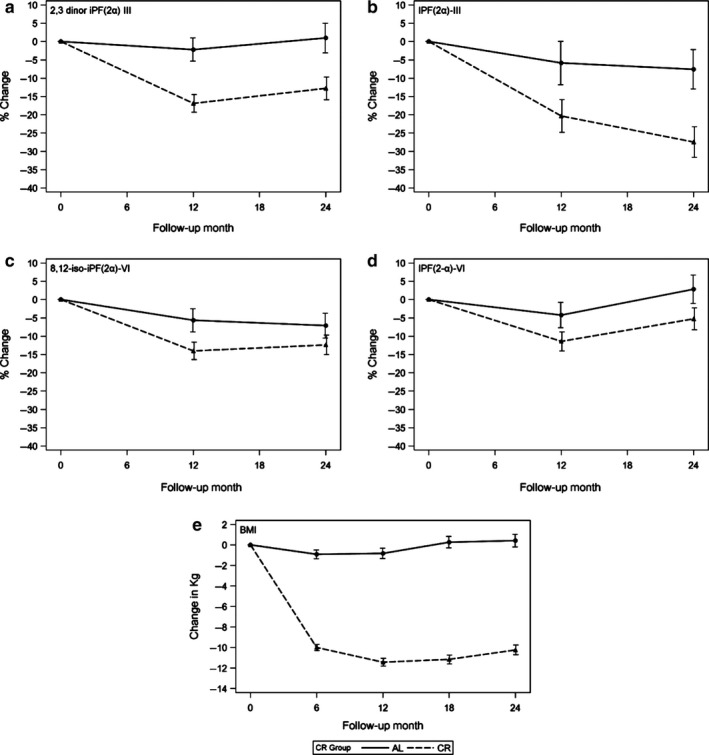
Change in urinary F2‐isoprostane concentrations and in body weight within CR and AL groups. Percent change in each F2‐isoprostane species as compared to the baseline level: (a) 2,3‐dinor‐iPF(2 α)‐III, (b) iPF(2α)‐III, (c) iPF(2 α)‐VI, (d) 8,12‐iso‐iPF(2α)‐VI, (e) body weight

F2‐isoprostanes are formed by nonenzymatic oxidation of arachidonic acid, and this process produces a mix of isomers with different positions of the oxygenated carbons—the III, IV, V, and VI series (Rokach et al., 1997). Therefore, in addition to the two F2‐isoprostanes from the III series, we measured two species from the VI series (which are less well studied, but abundant and potentially more sensitive markers of oxidative status (Pratico, Lawson, Rokach & FitzGerald, 2001; Dentchev, Yao, Pratico & Dunaief, 2007)). The adjusted effects of caloric restriction on VI series species were weaker than on III series (Table 3, Figure 1). There was no treatment‐by‐time interaction in any examined measure of F2‐isoprostanes. The general pattern of changes in urinary F2‐isoprostane concentrations was similar to weight changes in the AL‐ and CR groups (Figure 1).

A strong correlation between the four measured F2‐isoprostane species was observed. To take advantage of this intercorrelation, we performed a factor analysis using all four F2‐isoprostane species. First, the four F2‐isoprostane variables at baseline were normalized (mean* *= 0, *SD* = 1). The eigenvalue for the best linear combination of the four F2‐isoprostanes was 3.22, indicating that the factor explained 80.4% of the variance in the variables; correlations were 0.80, 0.77, 0.92, 0.72 between the factor and the four F2‐isoprostanes. That is, the summary scale contained 80.4% of the information contained in the four F2‐isoprostane markers. As no additional factor had an eigenvalue greater than 1, we chose to model a single factor, called isoprostane factor. By definition, isoprostane factor scores had a mea*n *= 0.0 and *SD* = 1 at baseline. Next, the isoprostane factor was defined for the two follow‐up points (12 months and 24 months), using the scoring and the normalization coefficients defined at baseline. Thus, any differences from 0 in the follow‐up levels of the factor should be interpreted as the proportion of a standard deviation value difference from the baseline mean. Testing of the intervention effect (CR vs AL) on changes in the isoprostane factor was conducted similar to the primary hypothesis (Table 3). For the AL group, there was approximately a 1/10^th^ *SD* decrease in the isoprostane factor (−0.098 *SD*), whereas the decrease in the CR group was (−0.358 *SD*) approximately 3.6‐fold greater (*p *= .004). This relative difference between the treatment groups increased to a 6.4 greater change at 24 months (−0.048 vs. −0.311 *SD*,* p *= .006) (Table 3). However, we did not observe a treatment‐by‐time difference in the effect (*p *= .97, for the time‐by‐treatment interaction).

### 2.1 Variables associated with F2‐isoprostane changes in the CR and AL groups

**Figure 2:**
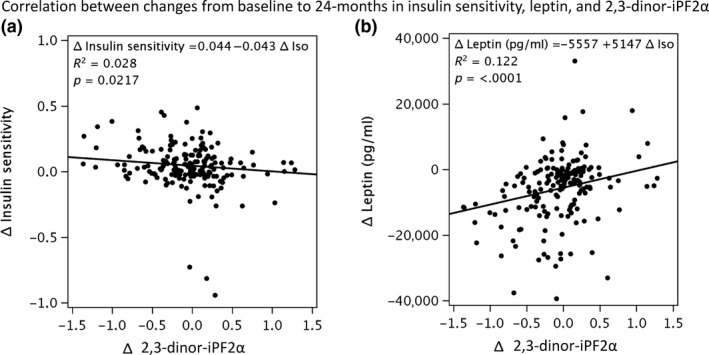
Correlations between changes in 2,3‐dinor‐iPF(2 α)‐III and two metabolic characteristics during the 2‐year intervention: (a) correlation with changes in plasma leptin levels and (b) correlation with changes in insulin sensitivity

At baseline, urinary 2,3‐dinor‐iPF(2α)‐III concentration was positively associated with fasting leptin (r_s_ = 0.476, *p *< .0001), HMW adiponectin (r_s_ = 0.342, *p *< .0001), C‐reactive protein (r_s_ = 0.274, *p *< .0001), fasting insulin (r_s_ = 0.159, *p *= .019), and triiodothyronine (r_s_ = 0.161, *p *= .018), and inversely with insulin sensitivity (r_s_ = −0.159, *p *= .019). However, in response to the intervention, only changes in leptin and insulin sensitivity were significantly correlated with changes in urinary 2,3‐dinor‐iPF(2α)‐III concentration (Figure 2). In particular, changes in leptin concentration were directly correlated with changes in urinary 2,3‐dinor‐iPF(2α)‐III concentration (r_s_ = 0.326, *p *< .0001 for both groups combined; r_s_ = 0.263, *p *= .0037 for CR group only). In contrast, change in urinary 2,3‐dinor‐iPF(2α)‐III concentration was inversely correlated with change in the insulin sensitivity (r_s_ = −0.234, *p *= .0012 for both groups combined; r_s_ = −0.193, *p *= .035 for CR group only).

## 3 DISCUSSION

Two years of CR in healthy, young and middle‐age nonobese individuals resulted in a significant and persistent reduction in urinary 2,3‐dinor‐iPF(2α)‐III and iPF(2α)‐III concentrations, reflecting lower accumulation of oxidative damage in multiple human tissues during the two‐year period. Urinary 2,3‐dinor‐iPF(2α)‐III concentrations were 17% and 13% lower after 1 and 2 years of CR, respectively. This reduction in urinary F2‐isoprostane concentrations in our CR volunteers was associated with a CR‐induced reduction in leptin and improved insulin sensitivity.

Whether or not oxidative stress plays a role in the biology of aging is still unclear (Perez et al., 2009); but, multiple lines of research indicate that oxidative stress is deeply implicated in the pathogenesis of several age‐related chronic diseases, including cardiovascular disease and cancer and it is considered a prevailing theory of human aging (Basu, 2008; Valko et al., 2016). In particular, obesity is associated with increased oxidative stress, and weight loss reduces urinary F2‐isoprostane levels in obese women (Davi et al., 2002; Duggan et al., 2016; Kanaya et al., 2011; Keaney et al., 2003). To the best of our knowledge, it is not known whether CR reduces F2‐isoprostanes in men, and most importantly in nonobese individuals. In our study, we observed that CR significantly reduced multiple F2‐isoprostane species in nonobese healthy men and women, and there was a significant correlation between change in serum leptin and urinary 2,3‐dinor‐iPF(2α)‐III concentrations.

It is well accepted that fat mass determines plasma leptin concentrations and that excessive adiposity is associated with elevated circulating leptin levels (Considine et al., 1996); this plays a key role in obesity‐induced oxidative stress (Milagro, Campion & Martinez, 2006). Injection of leptin significantly increases urinary F2‐isoprostanes in mice, probably by increasing the production of reactive intermediates such as H_2_O_2_ and hydroxyl radical through NOX activation (Beltowski, Wojcicka & Jamroz, 2003; Fortuno et al., 2010). Moreover, leptin may reduce the activity of the cellular antioxidant paraoxonase‐1 (PON‐1), which in turn increases oxidative stress (Beltowski et al., 2003). Consistent with these previously published findings, we observed a significant correlation between the change in plasma leptin concentration and the change in urinary 2,3‐dinor‐iPF(2α)‐III concentration.

The decline of insulin resistance, a key risk factor for several common cancers, may also mediate CR‐related reduction in oxidative stress (Longo & Fontana, 2010). Insulin resistance induced by excessive adiposity may increase the risk of developing cancer because of compensatory hyperinsulinemia and increased bioavailability of IGF1 (due to reduced IGFBP‐1 production), thereby up‐regulating the PI3K/AKT pathway and stimulating cell proliferation and genomic instability (Longo & Fontana, 2010). AKT activation inhibits the nuclear translocation of FOXO, resulting in a downregulation of DNA repair (i.e., DDB1) genes, and in an increase in cell proliferation (Cyclin D) and multiple antioxidant genes, such as SOD2 and catalase (Webb & Brunet, 2014). Accordingly, we found in this study a correlation between fasting insulin and urinary 2,3‐dinor‐iPF(2α)‐III concentration at baseline. Further, there was a significant inverse correlation whereby reductions in urinary 2,3‐dinor‐iPF(2α)‐III concentrations corresponded with improved insulin sensitivity.

The CR diet intervention did not mandate a particular dietary composition. Rather participants could self‐select their own diet, with dietary intake likely to vary throughout the trial in order for participants to remain adherent to the weight loss trajectory that fostered the desired level of CR. As changes in diet composition were not uniform across participants, it is unlikely that dietary changes can explain the observed reduction in F2‐isoprostanes.

The strengths of this work include the RCT design, highly structured and supervised 2‐year CR intervention in young and middle‐aged healthy normal weight or slightly overweight men and women, and comprehensive assessments of body composition and oxidative stress throughout the intervention. These strengths point to the observed responses being related to basic biological functions and not to those necessarily related to disease. These observations are also consistent with the marked improvements in cardiometabolic risk factors, including systolic and diastolic blood pressures, triglycerides, total cholesterol and glucose control (HOMA‐IR) observed in the CALERIE study (Ravussin et al., 2015). The consistency of the trends across all four F2‐isoprostane species led us to the conclusion that the overall oxidative status measured by these biomarkers can be summarized by an integrated function, the isoprostane factor. The high correlations between the isoprostane factor and each of the F2‐isoprostane species (.72 ≤ *r* ≤ .90) suggest that the four examined F2‐isoprostane species reflect the biological processes are closely related. However, caloric restriction induced greater changes in the III series species, indicating that F2‐isoprostane III series appear to be more sensitive to CR intervention than the VI series. It could be that metabolic pathways affected by CR favor formation of the III series F2‐isoprostane isomers.

In conclusion, data from this large randomized clinical trial showed that even in young and middle‐aged healthy lean or slightly overweight men and women, sustained CR significantly reduces oxidative stress, as reflected in lower urinary concentration of multiple F2‐isoprostane series. More studies are needed to understand the biological implications of reducing the accumulation of molecular damage leading to age‐associated chronic diseases, and whether or not other less drastic nutritional interventions in humans (e.g., intermittent fasting, moderate protein, or essential amino acid restriction) may mimic these effects. Of note, individuals in this study self‐selected their CR diet. Thereby, future CR studies that help participants to consume foods which provide antioxidant benefits may realize a greater benefit on markers of oxidative stress. The future implications of this work on long‐term aging remain to be studied.

## 4 EXPERIMENTAL PROCEDURES

### 4.1 Participants and study design

The rationale and study design of CALERIE Phase 2 have been previously described (Rochon et al., 2011). CALERIE 2 was a two‐year randomized controlled trial (RCT) that recruited healthy volunteers to receive an intervention designed to either reduce energy intake by 25% (CR group) or to maintain intake on an *ad libitum* basis (AL group). Eligibility criteria included men 20–50 years of age (inclusive) and women 21–47 years of age (inclusive) and required participants to be normal weight or slightly overweight (22.0 ≤ BMI<28.0 kg/m^2^) at the screening visit. A comprehensive screening procedure identified volunteers who were healthy to participate in a two‐year RCT of caloric restriction and adhere to the rigors of the study. Participants were randomized to 25% caloric restriction (CR) or *ad libitum* control (AL) in a 2:1 ratio; randomization was stratified for site, sex and BMI. Study interventions: From day 1, the CR intervention targeted a sustained 25% restriction of energy intake prescribed on the basis of the energy requirements determined from two, 2‐week doubly labeled water measures at baseline (Rickman et al. 2011; Redman et al. 2014). Adherence to 25% CR was fostered by regular group and individual meetings with trained interventionists, and a predicted weight loss trajectory was used as a guide to keep participants on track. Participants randomized to the control group were advised to continue their current diets on a completely ad libitum basis. No specific level of physical activity was required or recommended for either group. All participants received a multivitamin (Nature Made Multi Complete, Pharmavite LLC, Mission Hills, CA, USA) and calcium supplement (1000 mg/d, Douglas laboratories, Pittsburgh, PA, USA) to foster nutritional adequacy of the self‐selected diets. The study was approved by the institutional review boards at Pennington Biomedical Research Center, Washington University, Tufts University, and Duke University. Study oversight was provided by a Data and Safety Monitoring Board and registered at ClinicalTrials.gov NCT00427193. Full details of the trial can be found at calerie.duke.edu.

### 4.2 Clinical outcome assessments

Subjects were followed throughout the course of the two‐year study at clinical visits at each respective study site according to a manual of procedures. Urine and blood samples were obtained during an inpatient stay, in the morning following an overnight fast. Samples were processed in real time and were stored frozen until assays were performed at the end of the study. Analytes were measured at the University of Vermont Core Laboratory. Leptin (Linco Panel B, catalog number HADK2‐61K‐B), high molecular weight (HMW) adiponectin (R&D ELISA, Catalog number DRP300), and high‐sensitivity C‐reactive protein (BNII Nephelometer, OQ1Y21, OQ1Y13) were measured using ELISA. Fasting insulin (Elecsys, Roche Diagnostics, Indianapolis, IN, USA) and triiodothyronine were measured using a chemiluminescent immunoassay in an Immulite analyzer. Matsuda insulin sensitivity index was a measure derived from fasting insulin.

### 4.3 Measurements of urinary F2‐isoprostanes and creatinine

Concentration of F2‐isoprostanes in urine was adjusted for urinary creatinine to account for different urine diluteness between individuals, which varies depending on how much liquids a person ingested and how much was reabsorbed in the kidneys (Levey et al., 2005). Four isomers of F2‐isoprostanes—iPF(2α)‐III, 2,3‐dinor‐iPF(2 α)‐III, iPF(2 α)‐VI, and 8,12‐iso‐iPF(2α)‐VI—and creatinine were quantified by liquid chromatography‐tandem mass spectrometry (LC‐MS/MS) on Shimadzu 20A series LC and Applied Biosystems API 4000 QTrap MS/MS instruments as previously described (Il'yasova et al., 2010). Based on creatinine measurements, the urine samples were diluted to 0.65 mg/ml creatinine, and samples with creatinine levels equal to or below this value were analyzed without dilution. Sample preparation included addition of internal standards [iPF(2α)‐III‐d4, 8,12‐iso‐iPF(2α)‐VI‐d11, iPF(2α)‐VI‐d4] and 10 μl 1M HCl; washing of samples (500 μl) with 1 ml hexane; extraction of the analytes by ethyl acetate/hexane mixture (3/1, v/v); evaporation of the liquid; and resuspension of the residue in 150 μl of a mixture containing 70% mobile phase A (0.1% formic acid in water) and 30% methanol. Using LC‐MS/MS, 100 μl of sample was injected into two solid‐core C18 columns (Phenomenex Kinetex C18, 150 × 4.6 mm) in series to achieve chromatographic separation of the F2‐isoprostane isomers. The mass spectrometer was operated in negative mode with the following MRM transitions (m/z): 353/193 [iPF(2α)‐III], 357/197 [iPF(2α)‐III‐d4], 325/237 [2,3‐dinor‐iPF(2α)‐III], 353/115 [iPF(2α)‐VI and 8,12‐iso‐iPF(2α)‐VI], 364/115 [iPF(2α)‐VI‐d11], and 357/115 [8,12‐iso‐iPF(2α)‐VI‐d4]. Calibration samples covering the expected range of concentrations were prepared by adding pure material into pooled human urine, injected before and after the patient samples. Lower limits of quantification (LLOQ >80% accuracy) were 0.007, 0.34, 0.25, and 0.12 mg/ml for iPF(2α)‐III, 2,3‐dinor‐iPF(2α)‐III, iPF(2α)‐VI, and 8,12‐iso‐iPF(2α)‐VI, respectively.

### 4.4 Statistical analyses

Baseline characteristics were compared using Wilcoxon–Mann–Whitney test and Fisher's exact test. Spearman correlations examined relationships between baseline characteristics and F2‐isoprostanes at baseline. The same statistical methodologies as used in the parent RCT analysis were applied (Rochon et al., 2011). Intention‐to‐treat (ITT) analyses were performed by including all available observations in the analysis (see CONSORT diagram, Supplemental Material). Similar to the impact in drug trials, in this context, use of ITT analysis is intended to generate group differences that are similar to that expected in a similar population, incorporating level of compliance. All statistical tests were conducted at a two‐tailed type I error level. Results were generated using SAS version 9.2 and higher.

#### 4.4.1 Primary analysis

Repeated‐measures analysis of covariance was applied with change from baseline as the dependent variable; treatment, time, and the treatment‐by‐time interaction were independent variables; site, sex, BMI stratum, and baseline values of the biomarkers were covariates. The primary hypothesis was tested by defining contrasts among the regression parameters; the predicted mean ± standard error (se) are the adjusted values from this model. Type I error was controlled using a gatekeeping strategy (Dmitrienko & Tamhane, 2007). Comparisons were only performed at α = 0.05 if significance was α = 0.05 at the higher level in the hierarchy; otherwise, Bonferroni's correction was applied by multiplying the nominal *p*‐value by number of tests (truncated at 1.0) (Wright, 1992).

#### 4.4.2 Exploratory analyses

Using methods similar to the primary hypothesis testing, the exploratory analyses examined associations between three F2‐isoprostane species iPF(2α)‐III, iPF(2α)‐VI, and 8,12‐iso‐iPF(2α)‐VI and caloric restriction. We observed a strong correlation between the four individual F2‐isoprostane species, indicating a redundancy in the information contained in the individual measures. To take statistical advantage of this intercorrelation, we performed a factor analysis to define a combined variable, or factor, containing this common information at baseline. The advantage of a single summary variable comprised of the information held in common by a set of variables is that it is more reliable than any one single indicator, and the analysis of this summary variable avoids type I error issues inherent in the testing of multiple outcomes. This isoprostane factor was analyzed in a manner similar to the analysis of the primary hypothesis. In addition, we explored the correlations among F2‐isoprostanes, metabolic factors, and hormones known to be affected by CR: leptin, adiponectin, C‐reactive protein, fasting insulin, triiodothyronine, and insulin sensitivity (Fontana, Klein, Holloszy & Premachandra, 2006; Fontana, Meyer, Klein & Holloszy, 2004; Fontana et al., 2010; Lecoultre, Ravussin & Redman, 2011; Longo & Fontana, 2010).

## AUTHOR CONTRIBUTIONS

The Corresponding Author attests that two co‐leading authors are Drs. Dora Il'yasova and Luigi Fontana. All the authors had access to the study data, take responsibility for the accuracy of the analysis, and had authority over manuscript preparation and the decision to submit the manuscript for publication. The content of this article is solely the responsibility of the authors.

## CONFLICT OF INTEREST

The authors hereby declare that there was no conflict of interest associated with this study.
