# Supplementary material for: Effects of 2 years of caloric restriction on oxidative status assessed by urinary F2‐isoprostanes: The CALERIE 2 randomized clinical trial
Source: Aging Cell. 2018 Feb 9;17(2):e12719. doi: 10.1111/acel.12719 (PMC5847862; doi:10.1111/acel.12719)
Supplement: Supplementary file 1 [file ACEL-17-e12719-s001.doc]

**CALERIE CONS*O*RT DIAGRAM**

**Allocation**

N=218

**Follow-Up**

**Baseline**

Randomized (n=220)

26 Lost to follow-up

-3 women became pregnant

-6 moved away from study site

-3 withdrawn for safety

-8 withdrew consent

-6 dropped out for personal and other reasons

25 % CR (n= 145 )

♦ Received allocated intervention (n=143)

♦ Dropped out after baseline testing (n=2)

4 Lost to follow-up

- 3 women became pregnant

- 1 withdrew consent

Discontinued intervention (give reasons) (n= )

Ad Lib (n=75)

♦ Received allocated intervention (n= 75)

238 Admits

18 Dropped During Baseline

♦  Withdrew Consent (n=5)

♦  Adverse Events (n=3)

♦  Other (n=10)
